# Bronchiectasis in the Last Five Years: New Developments

**DOI:** 10.3390/jcm5120115

**Published:** 2016-12-08

**Authors:** Jun Keng Khoo, Victoria Venning, Conroy Wong, Lata Jayaram

**Affiliations:** 1 Department of Respiratory Medicine, Western Health, Melbourne 3011, VIC, Australia; gregkhoo@hotmail.com; 2 Department of Respiratory Medicine, Prince of Wales, Sydney 2031, NSW, Australia; venningv@gmail.com; 3 Department of Respiratory Medicine, Middlemore Hospital, Auckland 2025, New Zealand; Conroy.Wong@middlemore.co.nz; 4 Melbourne Medical School Western Precinct, The University of Melbourne, Melbourne 3021, VIC, Australia

**Keywords:** bronchiectasis, severity scores, microbiome, comorbidities, treatment

## Abstract

Bronchiectasis, a chronic lung disease characterised by cough and purulent sputum, recurrent infections, and airway damage, is associated with considerable morbidity and mortality. To date, treatment options have been limited to physiotherapy to clear sputum and antibiotics to treat acute infections. Over the last decade, there has been significant progress in understanding the epidemiology, pathophysiology, and microbiology of this disorder. Over the last five years, methods of assessing severity have been developed, the role of macrolide antibiotic therapy in reducing exacerbations cemented, and inhaled antibiotic therapies show promise in the treatment of chronic *Pseudomonas aeruginosa* infection. Novel therapies are currently undergoing Phase 1 and 2 trials. This review aims to address the major developments within the field of bronchiectasis over this time.

## 1. Introduction

Bronchiectasis unrelated to CF has until recently been an under-recognised and under-researched disorder associated with considerable morbidity and mortality. Characterised by cough and purulent sputum that cannot be cleared effectively, it is associated with, and mainly due to, frequent infections, leading to a vicious cycle of ongoing exacerbations, airway inflammation, dilatation, and damage. Treatment options are limited and involve respiratory physiotherapy to clear sputum and antibiotics to treat acute infections [1,2]. While accurate worldwide data on the prevalence of bronchiectasis is lacking, a recent review provides estimates [3], with the highest prevalence noted in the Asia Pacific region at 1,499,268 individuals in 2012 and expected to burgeon to 1,956,666 by 2020. An expert Australasian Task Force on bronchiectasis has highlighted the high prevalence of this disorder nationally especially within indigenous populations: in Central Australian Indigenous children, prevalence remains at 147 per 10,000 persons, with high rates also documented amongst Maori and Pacific Island children in New Zealand [1] and in Native American Indians [4]. Worldwide and Australasian data confirms the significant morbidity and health care burden associated with this disorder. There is a disproportionate health burden of bronchiectasis in the socioeconomically disadvantaged [5]. Admission to hospital for an exacerbation is associated with high readmission and increased mortality rates [6]. In our hospitals, readmission rates following an infective exacerbation of bronchiectasis in a calendar year range from 46% [6] to 61% with socioeconomic deprivation greatly contributing [6]. Over the last five years, the profile of bronchiectasis has increased due to the expanding availability of high-resolution CT scanning and dedicated research. This review aims to address the recent major developments within the field of bronchiectasis.

## 2. Natural History/Epidemiology

Research over the last decade has clearly identified factors associated with or predictive of a poor prognosis and disease progression in bronchiectasis. These include older age, poor lung function, and colonisation with *Pseudomonas aeruginosa* [7,8,9]. A recent meta-analysis pooling over 21 observational cohort studies comprised of 3683 subjects confirmed that *P. aeruginosa* colonisation is associated with increased mortality (odds ratio (OR): 2.95; 95% confidence interval (CI): 1.98–4.40; *p* < 0.0001) increased morbidity (exacerbations: mean difference, 0.97/year; 95% CI: 0.64–1.30; *p* < 0.0001;) and hospital admissions: (OR: 6.57; 95% CI: 3.19–13.51; *p* < 0.000) [10].

Investigations for underlying aetiologies of bronchiectasis remain an integral part of bronchiectasis management. While in many patients the disease is deemed idiopathic or post-infective, bronchiectasis is associated with chronic obstructive pulmonary disease (COPD), connective tissue diseases, and immunodeficiency [11,12]. It is well documented in the literature that COPD-related bronchiectasis is associated with more severe disease [11,13], as is rheumatoid arthritis-related bronchiectasis [14]. More recently, the existence of asthma has been associated with an independent increase in risk of bronchiectasis exacerbation [15] (refer to section: co morbidities).

## 3. Microbiological Developments

The relationship between exacerbation frequency and potential pathogenic microorganisms (PPM) other than *P. aeruginosa* either singly or in combination remains unclear and requires further investigation. Rogers and colleagues demonstrated that, while *P. aeruginosa* colonisation is associated with frequent exacerbations, *Haemophilus influenzae* (the predominant organism cultured in patients with bronchiectasis) is associated with a lower exacerbation rate on retrospective analysis of 107 sputum samples by culture and prospective examination of 42 sputum samples by lung microbiome analysis (see below) [16]. In contrast, McDonnell and colleagues in a retrospective study demonstrated similar exacerbation rates with culture positive *H. influenzae* and *P. aeruginosa* sputum samples (*n* = 155) [17].

### The Lung Microbiome

The organisms implicated with bacterial colonisation and recurrent infections in bronchiectasis are normally detected by growth on culture of airway fluid samples such as sputum or bronchial washings. Research utilising gene sequencing has explored alternative and more sensitive methods to culture-based methods of assessing lung microbiology or the “lung microbiome”. Culture independent methods offer better identification and quantification of microbial constituents of sputum than standard culture [16,18,19]. Sputum samples have been reported to detect only 83% of aerobic and anaerobic organisms, of those detected by gene pyrosequencing [18].

The lung microbiome is normally comprised of a diverse range of organisms, and the stability of the community is maintained by net immigration, via invasion of the airway mucosa, by inhalation and micro-aspiration, and by clearance through the inflammatory response [19]. In bronchiectasis, there is disruption and alteration or dysbiosis to the microbiome, precipitated by change within the local environment in factors such as temperature, glycosylation, and oxygenation. The two most common pathogens identified by gene sequencing are *H. influenzae* and *P. aeruginosa.* Other species identified within the microbiome have included *Veillonella* spp., *Prevotella* spp., *Streptococcus*, *Neisseria*, and less frequently *Stenotrophomonas maltophilia*, *Burkholderia cepacia*, *Moraxella catarrhalis*, and *Staphylococcus aureus* [16,18,19]. Gene sequencing facilitates the dectection of relative abundances, community structure, and diversity of the bacterial taxon [19]. Reduced diversity and predominance of *P. aeruginosa* (similar to sputum culture) is associated with more frequent exacerbations, reduced FEV_1_, and more severe disease as measured by the Bronchiectasis Severity Index [16,20]. Interestingly, *Veillonella* predominance, as detected by gene sequencing, has been associated with an increased risk of exacerbations [16]. Given *Veillonella* species are not routinely isolated on culture, they are likely to be underreported, suggesting a future role for microbiome analysis in prognostication and management.

The structure of microbial communities is highly individual between patients, and may relate weakly to disease state or antibiotic exposure [21]. The administration of prophylactic treatment with oral erythromycin (macrolide) therapy for one year, for example, demonstrated a decrease in *H. influenzae* and an increase in *P. aeruginosa* communities by lung microbiome analysis in patients without culture proven *P. aeruginosa* at baseline [22]. In this regard, concern may be raised for a bacterial shift towards more antibiotic-resistant organisms. In contrast, Tunney and colleagues in a small study (14 paired sputum samples) demonstrate that bacterial load and composition remain comparatively stable and diverse within the lung microbiome prior to and following antibiotic treatment for an exacerbation [18]. While demonstrating promise, lung microbiome analysis currently remains a research tool, and further trials are required and underway to assess its clinical relevance [18] and utility, especially in assessing early antibiotic resistance.

## 4. Phenotyping Bronchiectasis: Clinical, Radiological, and Microbiological Features

Similar to other airway disorders such as asthma and COPD, there is an increasing body of literature, suggesting that phenotyping patients by combining clinical, radiological, inflammatory, and microbiological characteristics allows identification of those individuals that have a more rapid disease progression or exacerbate frequently [23]. Those with *P. aeruginosa* in their sputum, low FEV_1_, and/or multi-lobe involvement on CT scan have a worse prognosis [24]. Aliberti and colleagues described four phenotypic clusters based primarily on microbiological analysis (the presence of *P. aeruginosa* or other infections) and the presence or absence of sputum by combining five European databases (Italy, United Kingdom, Ireland, Belgium, and Greece, *n* = 1145):

- Cluster 1: Chronic infection with *P. aeruginosa*;
- Cluster 2: Other chronic infection;
- Cluster 3: Daily sputum;
- Cluster 4: Dry bronchiectasis.

They found that those with infection (Clusters 1 and 2) had significantly more exacerbations, poorer lung function, poorer quality of life as measured by the St. George’s Questionnaire, and increased markers of airway inflammation (sputum neutrophil elastase, myeloperoxidase activity, and interleukin-1β levels) compared with Clusters 3 and 4. One-year mortality was similar in all groups, but three-year mortality was significantly higher in the *P. aeruginosa* group (Cluster 1, 14.5% at three years compared with 7.4% in Cluster 4). Martinez-Garcia and colleagues from Spain have recently identified four alternative clinical phenotypes with cluster analysis based on age, gender, disease severity, and exacerbations, *n* = 468:

- Cluster 1: Younger women with mild disease;
- Cluster 2: Older women with mild disease;
- Cluster 3: Older patients with severe disease and frequent exacerbations;
- Cluster 4: Older patients with severe disease and without frequent exacerbations.

Mortality was lower in the younger cohort of patients (16 deaths over five years in Clusters 1 and 2) compared with the older group with either frequent exacerbations or more severe disease (76 deaths over five years in Clusters 3 and 4). Further research needs to be undertaken in identifying phenotypes within other global populations, and especially within indigenous populations where the morbidity and mortality is disproportionately increased. From such research, some unifying characteristics allowing standardisation of clinical phenotypes globally in bronchiectasis might emerge.

### Inflammatory Phenotyping in Bronchiectasis

Bronchiectasis is associated with neutrophilic airway inflammation as measured by sputum and bronchoalveolar lavage differential cell counts, and neutrophil-specific by-products such as blood and sputum interleukin-8 and neutrophil elastase. Recent studies have identified specific profiles of mucins (proteins secreted by airway epithelium and found in sputum), airway disorders such as COPD, and bronchiectasis, with high levels of MUC2 gene found in the latter condition [25,26]. Mucins described in bronchiectasis (MUC2) may reflect bacterial load and *P. aeruginosa* colonisation and bronchiectasis severity as measured by the BSI (MUC2 and MUC5AC) [26], suggesting a potential future role in incorporating these by-products in characterising phenotypes.

## 5. Co-Morbidities and Bronchiectasis: COPD and Asthma

The prevalence of radiological bronchiectasis in patients with a primary diagnosis of COPD due to smoking varies from 4% in the ECLIPSE cohort [27] to 58% reported by Martinez-Garcia et al. [28]. The reverse, namely, the prevalence of clinical and radiological smoking-related COPD in patients with a primary diagnosis of bronchiectasis is low. Recent systematic reviews estimate the occurrence of COPD in patients with a predominant diagnosis of bronchiectasis between 3.9% and 15% [11,12]. Coexisting COPD and bronchiectasis, alternatively named the bronchiectasis and COPD overlap syndrome (BCOS), is associated with poorer outcomes [13]. Morbidity is increased with more frequent exacerbations and more PPM isolated on sputum culture (OR: 3.59; 95% CI: 1.3–9.9; *p* = 0.014) compared with COPD alone [28,29], and four-year mortality is increased (hazard ratio: 2.54; 95% CI: 1.16–5.56; *p* = 0.02) [13,28,30].

Recent literature suggests that asthma coexisting with bronchiectasis is also associated with more frequent exacerbations compared with bronchiectasis alone Mao and colleagues found that patients with bronchiectasis and asthma had a 2.6-fold increased risk of experiencing an exacerbation than those without associated asthma [15]. Another study found that, where asthma and COPD occur together (the asthma and COPD overlap syndrome (ACOS)), there are increased rates of bronchiectasis compared with asthma or COPD alone [31]. However, the effects of ACOS and bronchiectasis coexisting, in terms of disease progression and exacerbation rates are unknown, and more research is required.

## 6. Screening and Assessment Tools for Bronchiectasis

In the last two years, questionnaires have been developed and validated in bronchiectasis to assess *disease-specific quality of life* and disease severity. The Bronchiectasis Quality of life (QOL-B) is a disease-specific outcome measure for patients with bronchiectasis. It evaluates the effects of bronchiectasis on patients’ symptoms and daily functioning, and includes 37 items on eight scales. For each scale, scores are between 1 and 100. Higher scores indicate better health-related quality of life. Thresholds for a clinically significant change in health status using *QOL-B* have been established [32].

Two scores have been developed to assess *bronchiectasis severity* (Table 1): the Bronchiectasis Severity Index (BSI) and the Forced expiratory volume in 1 s (FEV_1_), Age, Colonisation with *Pseudomonas aeruginosa*, Radiological extent, and Dyspnoea (FACED) score [33,34]. Both have been validated and include clinical, radiological, sputum microbiology and spirometric criteria; specifically age, FEV_1_ percent predicted, radiological extent of bronchiectasis, *P. aeruginosa* colonisation, and measures of breathlessness (dyspnoea). The BSI also evaluates Body Mass Index, previous hospitalisation with an exacerbation, and the number of exacerbations in previous year. Both the FACED and BSI predict mortality, while the BSI additionally predicts morbidity, including risk of hospitalisation and impaired quality of life. A recent comparison (*n* = 1612) of both severity scores in 7 European cohorts found that FACED demonstrated a higher specificity (area under receiver operator characteristic curve (AUC) 93% vs. 70%), while the BSI was more sensitive (65% vs. 28%) for predicting mortality from bronchiectasis [35]. Both scores accurately predicted five-year mortality AUC 0.79 for BSI and 0.8 for FACED [36]. The FACED score had superior predictive power for 15-year mortality compared with BSI (AUC 0.82 vs. 0.69, *p* = 0.05) [36]. Further studies are required to assess responsiveness to interventions.

## 7. Databases and Registries for Bronchiectasis

Over the last five years, there has been a recognition that clinicians and researchers need to work collaboratively to progress the understanding of the pathophysiology of bronchiectasis and develop new treatments for this disorder. To that end, a number of collaborative initiatives have been established. The largest is the European Multicentre Bronchiectasis Audit and Research Collaboration (EMBARC) registry created in 2012 (www.bronchiectasis.eu). It aims to enrol 10,000 patients with non-CF bronchiectasis throughout Europe over the next five years and to follow them longitudinally. Baseline data and annual follow-up data will be collected on demographics, co-morbidities, aetiology, medications, microbiology, exacerbations, and health utilisation. The severity, prognosis, and survival of bronchiectasis will be ascertained. It will also provide a means of trialling new treatments. Similar registries have been initiated in other countries including America in 2007 and Australia in 2016.

## 8. Treatments for Bronchiectasis

### 8.1. Non-Pharmacological

#### 8.1.1. Airway Clearance Techniques (ACTs)

Clearance of sputum actively using ACTs is advocated and regularly implemented to reduce the risk of exacerbations in bronchiectasis. Techniques include postural drainage, an active cycle of breathing and other selected breathing manoeuvres, positive expiratory pressure devices, airway oscillating devices, and high-frequency chest wall oscillation. There is, however, little objective evidence documenting the benefit of ACTs with a 2015 Cochrane collaboration finding seven small (sample size of less than 30 participants) and short-term (less than or equal to six months) randomised, predominantly crossover, studies in the area. As these studies were heterogeneous, data could not be pooled. Nevertheless, the studies indicated that ACTs either alone or in combination were safe and had a minor and variably significant benefit on symptoms, quality of life, lung function, and sputum clearance [37]. Large randomised controlled studies of longer duration are required to determine the effect of ACTs on clinically relevant outcomes, such as frequency of exacerbations and antibiotic use [37].

#### 8.1.2. Exercise

A recent meta-analysis (n = 4 studies, 164 participants) demonstrated that a supervised outpatient exercise or pulmonary rehabilitation programme (comprising of both exercise and education) over eight weeks improved the incremental shuttle walk distance (weighted mean difference (WMD) = 67 m; 95% confidence interval (CI), 52–82 m) and quality of life (SGRQ WMD = −4.65; 95% CI: −6.7 to −2.6 units). These clinically significant improvements were noted immediately following the intervention but were not sustained at six months. Fewer exacerbations over 12 months were noted with regular exercise (median, 2 vs. 1; p = 0.013) [38].

### 8.2. Pharmacological

#### 8.2.1. Macrolide in Non-CF Bronchiectasis

A major advance in the management of bronchiectasis is the use of prophylactic macrolide therapy to prevent exacerbations via antibacterial and anti-inflammatory immunomodulatory pathways [39]. Three key randomised placebo controlled studies in adults and one in children with the acronyms EMBRACE, BAT, BLESS, and BIS respectively, published in close succession, examined the clinical benefit of prophylactic azithromycin and erythromycin in adults with bronchiectasis [40,41,42,43]. They demonstrated a pooled reduction in exacerbation frequency of nearly 60% with macrolide therapy (risk ratio: 0.42 (0.29, 0.61) [44] and increased time to first exacerbation, cementing their role in this disorder [45] (Table 2). A small (*n* = 52) open label parallel trial comparing 150 mg/day roxithromycin to a placebo over six months found that roxithromycin significantly increased time to first exacerbation (median 264 vs. 113 days, *p* = 0.022), reduced sputum biomarkers of airway inflammation, and improved CT-based measures of airway thickness [46]. Type of macrolide used, dose, and duration of therapy varies amongst studies, making direct comparisons difficult. Although the number of exacerbations are significantly reduced in patients maintained on macrolides, the number of patients with severe exacerbation requiring admission into hospital is not significantly reduced [43]. The beneficial effect of macrolides on lung function and health-related quality of life measures (St. George’s Respiratory Questionnaire) from randomised controlled trials (RCTs) are small and of debatable clinical significance (Table 2), while pooled data that include open labelled studies describe greater improvements [43,45]. Significantly increased adverse events with macrolides compared to a placebo include diarrhoea and abdominal discomfort [43]. Other common antibiotic side effects of nausea or vomiting, headache, and rash are not significantly increased.

There has been a theoretical risk of cardiac arrhythmias and cardiac arrest and sensorineural hearing loss given the class effect of macrolides on prolongation of the QT interval and ototoxicity. However, increased frequency of these adverse effects compared to the placebo has not been noted in the studies to date [40,41,42]. Only the BAT trial systematically examined the adverse event of auditory decline based on self-reported hearing impairment or tinnitus. There was no difference between azithromycin and placebo groups [41].

The BLESS trial examined cardiac events and did not demonstrate a significant difference in the QTc interval or any new cardiac arrhythmia between erythromycin and placebo groups [42]. In 2012, Ray and colleagues retrospectively reviewed azithromycin use and the risk of cardiovascular death in an American (Tennessee) cohort of patients who were prescribed azithromycin (347,795 prescriptions). They were matched to those not on antibiotics and to patients who were prescribed amoxicillin, ciprofloxacin, or levofloxacin [47]. The authors found a significantly increased risk of cardiovascular death with five days of treatment with azithromycin compared with the placebo (hazard ratio: 2.88; 95% CI: 1.79 to 4.63; *p* < 0.001), amoxicillin (hazard ratio: 2.49; 95% CI: 1.38 to 4.50; *p* = 0.002), and ciprofloxacin and estimated 47 additional deaths per 1 million courses. Since then, several studies have re-visited this issue and have suggested the actual risk is lower. A Danish cohort study found an increase in the risk of death with azithromycin when compared with the placebo (rate ratio (RR): 2.85; 95% CI: 1.13 to 7.24) but not with penicillin V (RR: 0.93; 95% CI: 0.56 to 1.55 [48]. A similar retrospective cohort study in Ontario in older adults (age > 65) receiving a new prescription for an oral macrolide antibiotic (azithromycin, clarithromycin, or erythromycin) found no increase in risk of ventricular arrhythmia (0.03% vs. 0.03%; relative risk 1.06, 95% CI: 0.83–1.36) or all cause mortality at 30 days, and which was actually lower, compared with non-macrolide antibiotics (0.62% vs. 0.76%; relative risk: 0.82, 95% CI: 0.78–0.86). A literature review of published studies to 2014 found that arrhythmogenicity was related to macrolide concentration in the blood, with oral dosing posing a low risk [49]. The incidence of arrhythmias and death with macrolides in the absence of underlying risk factors such as QT prolongation and cardiac comorbidities is low [49,50]. Nevertheless, a baseline ECG to assess QT prolongation prior to commencing macrolides is easy to acquire and is recommended.

The major concern with prophylactic macrolide use is the evolution of macrolide resistance [51]. This was prospectively assessed in the BAT and BLESS trials and found to be markedly increased compared with placebo (88% organisms resistant to AZT in sputum samples compared with 26% in the placebo group in the former study and 27.7% of streptococci isolates from oropharyngeal swabs in the erythromycin group were resistant compared with 0.04% in the placebo group *p* < 0.0001 in the latter) [41,43]. Hare et al. showed that, in Australian indigenous children, macrolide resistance was determined by macrolide adherence. Increased adherence (≥70%) was associated with an overall lower pathogen carriage rate; this translates into overall lower resistance rates. However, *S. aureus* specifically remained 100% macrolide-resistant likely due to different mechanism for adopting resistance [52]. Despite concerns regarding macrolide resistance, it is unclear if microbiological macrolide resistance translates into adverse clinical outcomes. The current clinical consensus is to consider prophylactic antibiotics in those patients with frequent (3 or more) exacerbations within the preceding year who have failed standard therapies [2,53].

#### 8.2.2. Inhaled Antibiotics

The role of prophylactic inhaled antibiotics in non-CF bronchiectasis is evolving with the aim of delivering the drug directly into airway to improve antibacterial efficacy and to reduce systemic side effects. Antibiotics trialled to date include the aminoglycosides (nebulised gentamicin/tobramycin), colistin, ciprofloxacin, and aztreonam (Table 3). These inhaled antibiotics have collectively demonstrated a reduction in sputum bacterial load; their effectiveness on clinical outcomes are awaited [54].

Murray and colleagues [55] demonstrated in a single-blind study (*n* = 65) that 12 months of treatment with nebulised gentamicin (80 mg) compared with normal saline twice daily reduced bacterial density, increased eradication of organisms, improved quality of life, increased six-minute walk distance, and increased the time to first exacerbation. No improvement was noted in lung function. A significant 22% (vs. 6%) of bronchospasm was documented, and all outcomes returned to baseline 3 months after treatment. Earlier studies with 300 mg of nebulised inhaled tobramycin given twice daily from 30 days for up to six months have demonstrated similar findings with a reduction in bacterial density in patients colonised with *P. aeruginosa*, and variable improvements in clinical outcomes associated with a significant increase in cough, wheeze [56,57], and bronchospasm [58], thereby requiring careful monitoring or precluding its use. More recently, free base dry powder tobramycin delivered by either the Cyclops dry powder device™ or Podhaler™ (rather than the powder formulation of tobramycin sulphate which contains excipients, or ingredients other than the active drug, that may cause potential adverse effects) is showing promise in Phase 1 and 2 trials [59].

Colistin, a polypeptide antibiotic effective against Gram-negative Bacilli, has demonstrated similar results. Patients (*n* = 144) colonised with *P. aeruginosa* received either 1 m IU or 0.45% saline twice daily for six months in a RCT study. The primary endpoint, median time to the first exacerbation, was similar in both groups by an intention to treat analysis 165 (42) vs. 111 (52) days in the colistin and placebo groups, respectively (*p* = 0.11). It was, however, increased in those adherent to therapy (168 days in the colistin group vs. 103 days with placebo, *p* = 0.038). Quality of life scores measured by the SGRQ improved significantly by 10.5 units, and bacterial density reduced in the active arm without an increase in adverse effects compared with placebo [60].

Nebulised dry powder inhalation of ciprofloxacin (RESPIRE 1) for 28 days resulted in a reduction of bacterial density and the eradication of bacteria in 27% of patients (*n* = 124) without significant side effects. Bacterial growth, however, returned once the active drug was stopped [60]. This has led to recent studies trialling cyclical inhaled antibiotics with alternative modes of antibiotic delivery to ensure bacterial suppression without an increase in bacterial resistance in chronic lung disease. To that end, antibiotic nanoparticles (nano-antibiotics) or antibiotics encapsulated into liposomes (a minute spherical sac of phospholipid molecules) and polymer nanoparticles as carriers are emerging as a new therapy to treat Pseudomonal infections. These antibiotics, through their encapsulation, theoretically penetrate through mucus and deposit deeper into the lungs. Liposomal ciprofloxacin, in a double-blind, Phase 2, RCT (ORBIT 2) given in a cyclical manner (3 cycles of 28 days on/28 days off; 6 months total) to 42 patients with *P. aeruginosa* sensitive to ciprofloxacin, reduced bacterial density significantly during active cycles. This returned to baseline off treatment, compared with the placebo. Time to first exacerbation also increased 134 vs. 58 days significantly compared with the placebo (*p* = 0.057; *p* = 0.046 per protocol) without an increase in side effects [61]. Building on this, the Phase 3 studies (ORBIT 3 and 4) with inhaled cyclical ciprofloxacin comprised of a mixture of liposome encapsulated and unencapsulated ciprofloxacin have just been completed. In these identical multicentre double-blind, placebo-controlled trials over 48 weeks, 278 patients in ORBIT 3 and 304 in ORBIT 4 with *P. aeruginosa* colonisation were enrolled. They received six cycles of 28 days ‘on treatment’ with ciprofloxacin or placebo, plus 28 days ‘off treatment’, followed by a 28 day open label period where all participants received ciprofloxacin. The primary outcome is the time to first pulmonary exacerbation with secondary outcomes including exacerbation frequency and improvement in the quality of life measures. Results are anticipated towards the end of 2016. Similarly, the results of the RESPIRE 2 study, a multi-centre, double-blind, placebo-controlled RCT comparing cyclical dry powder-inhaled ciprofloxacin 32.5 mg BID administered for 28 days on/28 days off or 14 days on/14 days, will be released shortly.

The effectiveness of cyclical nebulised aztreonam, a monobactam antibiotic used to treat Gram-negative infections, was assessed in the AIRBX1 and AIRBX2 studies: two parallel double-blind, Phase 3 RCTs (*n* = 266 and 274). Aztreonam 75 mg TDS was given via eFlow nebuliser over 2 treatment cycles of alternate 28 days “on” and 28 days “off” for 4 months total. The primary outcome was clinical: quality of life measured by the Quality of Life-Bronchiectasis Respiratory Symptoms scores (QoL-B-RSS) at the end of the four-month period. This did not achieve clinical nor statistical significance and exacerbation frequency was not reduced. Similar to other studies, a greater reduction in sputum Gram-negative bacterial density was noted during the “on” phase, which increased towards baseline during “off”-treatment periods. In both studies, adverse effects of dyspnoea, cough, and increased sputum production were significantly increased in the treatment arm [63].

Further studies are needed to examine the optimum duration of inhaled antibiotics, cyclicity, and their effectiveness in improving clinical outcomes.

#### 8.2.3. Inhaled Hyperosmolar Agents, and Inhaled and Ingested Mucolytic Agents

Inhaled hyperosmolar agents include hypertonic saline and mannitol, which, with their high osmolarity, mechanistically draw fluid and mucus into the airway-enhancing clearance. Small trials have shown equivocal benefit with hypertonic saline on clinical outcomes, such as exacerbation rate, quality of life, and lung function, and larger studies are required [64]. Inhaled mannitol at 400 mg twice daily given for one year in 461 patients did not reduce exacerbation rate, the primary outcome, compared with the control group. A small improvement was noted in secondary outcome measures, increased time to first exacerbation (HR 0.78, *p* = 0.022) and quality of life (SGRQ: −2.4 units, *p* = 0.046) without side effects. The role of mannitol is thus currently limited to selected patients. [65]. A Cochrane review recently updated its established 2001 appraisal of mucolytic therapy in bronchiectasis. No recent studies were found, and the four studies previously included were heterogeneous, precluding a meaningful cohesive analysis [66]. Nevertheless, ingested bromohexine and erdosteine in small studies improved sputum clearance with variable improvements in lung function, and larger trials are warranted. Inhaled recombitant human deoxyribonuclease (RhDNase) increases frequency of exacerbations and worsens lung function in non-CF bronchiectasis and should be avoided [66].

#### 8.2.4. Other Anti-Inflammatory Agents

##### Neutrophil Elastase Inhibitors

Neutrophil elastase (NE), a protease released by neutrophils in bronchiectasis as part of the inflammatory response contributes to airway and lung parenchymal damage and mucus secretion. Inhibiting neutrophil elastase may potentially dampen the neutrophilic inflammatory response and reduce exacerbations, improving symptoms and lung function in patients with bronchiectasis. In a Phase 2 randomised, double-blind placebo-controlled, parallel group, signal-searching study, Stockley and colleagues investigated the efficacy and safety of AZD9668 (NE inhibitor) at 60 mg twice daily over four weeks in 38 patients [67]. The baseline characteristics of the groups were not entirely matched. There were more males and, importantly, more use of inhaled long-acting muscarinic antagonists (LAMAs), long-acting beta-agonists/inhaled corticosteroids (LABAs/ICS’s), and azithromycin in the AZD9668 group. There was no difference in the primary outcome, the absolute and percentage neutrophil counts in morning (waking and post-waking) sputum samples, between treatment and placebo groups. In terms of clinical outcomes, AZD9668 showed a statistically significant improvement in FEV_1_ of 100 mL (*p* = 0.006) and 130 mL in slow vital capacity compared with the placebo. However, due to the imbalance in baseline characteristics, it is difficult to know whether this improvement could be, in part, due to the greater usage of inhaled bronchodilators in this group. Quality of life (SGRQ-C scores) was similar in both groups. Sputum inflammatory biomarkers, IL-6, Regulated on Activation, Normal T-cell Expressed and Secreted (RANTES), and plasma IL-8, improved significantly with treatment. Major adverse events were not a feature.

##### Statins

In vitro studies have demonstrated that statins decrease airway eosinophilia, neutrophilia, and remodelling [68]. Mandal and colleagues performed a proof-of-concept RCT to establish if atorvastatin could reduce cough and markers of inflammation in patients with bronchiectasis [69]. Sixty stable patients were randomised to receive atorvastatin at 80 mg twice daily or a placebo for six months. Patients colonised with *P. aeruginosa* and/or on long-term oral macrolides were excluded. Inhaled corticosteroid use was permitted. The primary outcome, a reduction in cough at six months assessed by the Leicester Cough Questionnaire (LCQ), was achieved. After adjustments for baseline smoking status, there was an improvement of 1.4 units in the LCQ (which has an established minimally clinically important difference of 1.3 units) in the atorvastatin group (*p* = 0.04). Quality of life assessed by the SGRQ mildly improved, but did not achieve statistical or clinical significance. There was a trend towards reduction in serum inflammatory markers C Reactive Protein and IL-8, while sputum neutrophil apoptosis increased with treatment, suggesting an anti-inflammatory effect. Increased adverse events (10 vs. 3) were noted with atorvastatin (diarrhoea, headache, and deranged liver function), leading to early discontinuation in five patients. Although promising, larger, multi-centre, adequately powered studies with more generalisable inclusion criteria are required to validate the above findings.

##### Chemokine Receptor 2 (CXCR2) Antagonists

Chemokines are signalling cytokines such as IL6 and IL8 that promote neutrophil migration as part of the airway inflammatory response. A recent randomised double-blind placebo-controlled multicentre Phase 2 study evaluated the effect of a chemokine receptor 2 antagonist AZD5069 on sputum neutrophil counts in 52 adults with bronchiectasis. Patients received AZD5069 at 80 mg or a placebo orally twice daily for 28 days. The primary outcome measure, a change in sputum absolute neutrophil count, fell significantly by 69% with AZD5069 compared to the placebo. Exacerbation frequency was similar in treatment and placebo groups (nine vs. eight), but more study discontinuations were noted with AZD5069 (four vs. zero). Sputum fluid phase markers including interleukin IL-8 were significantly increased with AZD5069 compared with the placebo, and the reasons for this are unclear [70].

## 9. Conclusions

Bronchiectasis has been considered an orphan disease for many years. Over the last decade, and especially over the five years and through the establishment of global networks of research, there has been considerable progress on many fronts: a further understanding of the pathophysiology and microbiology, the methods of assessment, and the development of new treatments and potential treatments undergoing Phase 2 and 3 trials. Future areas for research have been identified [71]. Effort, however, needs to be directed to reducing the burden of disease and the factors that drive this in indigenous and low socioeconomic populations where the disease is most prevalent.

## Figures and Tables

**Table 1:** Comparing the Bronchiectasis Severity Index (BSI) and FACED severity scores in bronchiectasis. Data adapted from the EMBARC registry, www.bronchiectasis.eu, ref. [34]. FEV_1_: Forced expiratory volume in 1 s; MRC: Medical Research Council; BMI: Body Mass Index.

Bronchiectasis Severity Index (BSI) Criteria	0 Point	1 Point	2 Points	3 Points	4 Points	5 Points	6 Points	FACED Bronchiectasis Severity Criteria	0 Point	1 Point	2 Points
FEV_1_ % predicted	>80%	50%–80%	30%–49%	<30%	-	-	-	FEV_1_ % predicted	>50%	-	≤50%
Age (years)	<50	-	50–69	-	70–79	-	>80	Age (years)	≤70	-	>70
Colonisation	No	Chronic colonisation with any organism	-	*P. aeruginosa* colonisation	-	-	-	Colonisation	No *P. aeruginosa*	Presence of *P. aeruginosa*	-
Radiology: extension	<3 lobes	≥3 lobes or cystic changes	-	-	-	-	-	Extension	0–2	>2 lobe	
Dyspnoea score (MRC)	1–3	-	4	5	-	-	-	Dyspnoea score (MRC)	1–2	3–4	-
BMI kg/m^2^	≥18.5	-	<18.5	-	-	-	-	-		-	-
Exacerbations in the last 12 months	0–2	-	≥3	-	-	-	-	-	-	-	-
Hospital admissions in the last 2 years	No	-	-	-	-	Yes	-	-	-	-	-
BSI Score: Mild bronchiectasis: 0–4 points: 1 year outcome 0%–2.8% mortality, 0%–3.4% hospitalisation rate 4 year outcomes 0%–5.3% mortality, 0%–9.2% hospitalisation rate Moderate bronchiectasis: 5–8 points: 1 year outcome 0.9%–4.8% mortality rate, 1%–7.2% hospitalisation rate 4 year outcomes 4%–11.3% mortality rate, 9.9%–19.4% hospitalisation rate Severe bronchiectasis: 9+ points: 1 year outcome 7.6%–10.5% mortality rate, 16.7%–52.6% hospitalisation rate 4 year outcomes 9.9%–29.2% mortality rate, 41.2%–80.4% hospitalisation rate								FACED Score: Mild: 0–2 points: 5-year mortality 4.3% Moderate: 3–4 points : 5-year mortality 24.7% Severe: 5–7 points: 5-year mortality 55.9%			

**Table 2:** Recent key macrolide RCTs in bronchiectasis.

Macrolide RCT	Regimen	*n*	Duration	Exacerbation Rate Ratio (95% CI)	QoL (SGRQ) Mean Difference (95% CI)	FEV_1_ (L or % Predicted) Mean Difference (95% CI)	Adverse Effects
Azithromycin	500 mg 3 times a/week; EMBRACE [40]	141	6 mths treatment, 6 mths followup	0.38 (0.26, 0.54); *p* < 0.0001	–3.25 (–7.21, 0.72); NS	0.04 L (−0.03, 0.12); NS	Mild GI *p* = 0.005
Adult	250 mg daily; BAT [41]	83	12	Hazard ratio = 0.29 (0.16, 0.51)	−2.06 (−11.1, 7.01); NS	−3.66 L (−14.78, 7.46); NS	Diarrhoea
Child	30 mg/kg once a week; BIS [43]	88	24	0.50 (0.35–0.71); *p* < 0.0001	NA	NA	NS
Erythromycin	400 mg ethylsuccinate twice daily; BLESS [42]						
Adult		117	12	0.57 (0.42, 0.77); *p* = 0.003	−5.3 (−12.6, 2.1); NS	2.2% predicted (0.1%, 4.3%); *p* = 0.04	NS
Roxithromycin Adult	150 mg daily; [46] Open label	52	6 mths	Delayed time to first exacerbation 264 vs. 113 days (*p* = 0.022)	NA	NA	Mild nausea

RCT: randomised controlled trial; *n*: number of subjects; CI: confidence interval; QoL: quality of life; SGRQ: St. George’s Respiratory Questionnaire; FEV1: forced expiratory volume in 1 s; L: litres; NS: not significant; GI: gastrointestinal.

**Table 3:** Key recent inhaled antibiotic adult RCTs in bronchiectasis.

Inhaled Antibiotic Adult RCT	Regimen & Delivery	*n*	Duration	Main Findings	Adverse Effects
Gentamicin	80 mg BD via jet nebuliser [55]	65	12 months	Greater reduction in sputum bacterial density (log_10_ CFU/g)	Broncho-Spasm
				Less sputum purulence	
				Greater exercise capacity	
				Fewer exacerbations	
				Increased time to first exacerbation	
				Greater improvements in Leicester Cough Questionnaire & SGRQ	
				No significant differences in 24 h sputum volume, FEV_1_, FVC, FEF	
Colistin	1 million IU BD via I-neb AAD system, administered within 21 days of completing a course of anti-pseudomonal antibiotics [62]	144	6 months	No significant differences in time to first exacerbation in ITT population Increased time to first exacerbation in adherent patients	NS
				Greater decrease in sputum *P. aeruginosa* density (log_10_ CFU/g)	
				Significant improvement in SGRQ	
				No significantly increased *P. aeruginosa* resistance	
				No significant differences in FEV_1_	
Ciprofloxacin	32.5 mg BD via dry powder inhalation (RESPIRE 1) [60]	124	4 weeks	Greater reduction in sputum bacterial density (log_10_ CFU/g)	NS
				Increased sputum pathogen eradication rate at the end of treatment	
				No significant differences in FEV_1_ % pred, FVC, SGRQ	
	Liposomal ciprofloxacin 150 mg + free ciprofloxacin 60 mg daily via PARI LC sprint nebuliser over 3 treatment cycles of alternate 28 days “on” and 28 days “off” (ORBIT 2) [61]	42	6 months	Greater decrease in sputum *P. aeruginosa* density (log_10_ CFU/g)	NS
				Increased time to first exacerbation	
				No significant differences in FEV_1_, SGRQ, 6MWT distance	
				Greater reduction in sputum Gram-negative bacterial density (log_10_ CFU/g), but increasing towards baseline during off-treatment periods	
Aztreonam	75 mg TDS via eFlow nebuliser over 2 treatment cycles of alternate 28 days “on” and 28 days “off” (AIRBX1 & 2) [63]	540	4 months	No difference in quality of life measured by Quality of Life-Bronchiectasis Respiratory Symptoms scores (QoL-B-RSS)	Dyspnea, Cough, Increased sputum
				No improvement in exacerbation risk	
				Higher MIC for aztreonam for target Gram-negative bacteria after 4 weeks	

RCT: randomised controlled trial; *n*: number of subjects; BD: twice daily; TDS: three times daily; CFU: colony forming units; SGRQ: St. George’s Respiratory Questionnaire; FEV_1_: forced expiratory volume in 1 s; FVC: forced vital capacity; FEF: forced expiratory flow; IU: international units; ITT: intention to treat; NS: not significant; 6MWT: 6 min walk test; MIC: minimum inhibitory concentration; A-AD: adaptive-aerosol delivery device.
